# Neural substrates of norm compliance in perceptual decisions

**DOI:** 10.1038/s41598-018-21583-8

**Published:** 2018-02-20

**Authors:** U. Toelch, A. Pooresmaeili, R. J. Dolan

**Affiliations:** 1 Berlin School of Mind and Brain, Humboldt Universität, Berlin, Germany; 2 Biological Psychology and Cognitive Neuroscience, Freie Universität Berlin, Berlin, Germany; 3 Berlin Institute of Health, Berlin, Germany; 4 Perception and Cognition Group, European Neuroscience Institute, Göttingen, Germany; 5 Wellcome Trust Centre for Neuroimaging, London, UK; 6 Max Planck UCL Centre for Computational Psychiatry and Ageing Research, London, UK

## Abstract

Societal norms exert a powerful influence on our decisions. Behaviours motivated by norms, however, do not always concur with the responses mandated by decision relevant information potentially generating a conflict. To probe the interplay between normative and informational influences, we examined how prosocial norms impact on perceptual decisions subjects made in the context of a simultaneous presentation of social information. Participants displayed a bias in their perceptual decisions towards that mandated by social information. However, normative prescriptions modulated this bias bi-directionally depending on whether norms mandated a decision in accord or contrary to the contextual social information. At a neural level, the addition of a norms increased activity in prefrontal cortex and modulated functional connectivity between prefrontal and parietal areas. The bi-directional effect of our norms was captured by differential activations when participants decided against the social information. When norms indicated a decision in line with social information, non-compliance modulated lateral prefrontal cortex activity. By contrast, when norms mandated a decision against social information norm compliance increased activity in the anterior cingulate cortex. Hence, social norms changed the balance between a reliance on perceptual and social information by modulating brain activity in regions associated with response inhibition and conflict monitoring.

## Introduction

Behaviour is strongly influenced by societal rules (i.e. norms) whose prescriptions often conflict with decision-relevant information acquired through integration of observational (social information) and trial and error learning (individual information). The integration of social and individual information has been studied at theoretical^1–3^, behavioural^4–6^, and neuronal levels^7–11^. Despite this, the impact of normative influences and their interaction with informational influences is underexplored. The influence of norms on decision making thus provides the primary focus for the current paper.

Informational and normative influences serve distinct goals. Informational influences reduce uncertainty about environmental parameters and improve response accuracy (*optimality goal*). Normative influences, in contrast, serve a more complex function that includes consolidation and maintenance of social relationships and an avoidance of societal retribution (*compliance goal*)^12–14^. A conflict between these two goals can lead to diverging behavioural responses and may form the basis for expression of individual differences that depend on factors such as group identification or moral conviction^15,16^, where these can have a long-lasting impact on behaviour and beliefs^17,18^. Whereas a distinction between informational and normative influences is widely accepted^12,19,20^ an interaction between them, despite its importance, is poorly understood^3^.

Here, we probe an interplay between informational and normative influences by exposing participants to optimality and compliance goals that can either be in accord or conflict. Players received money for deciding correctly in a perceptual task, where they were also provided with social information (the estimates of two additional players). Under an optimality goal, players should seek to maximise the number of correct answers. We build on an extensive literature showing that subjects balance individual (in this case perceptual) and social information to optimise their behaviour^7,8,21,22^. Additionally, we introduce two norms that prompt subjects to decide either *with* or *against* a majority decision (i.e. social information). Both norms serve the same prosocial compliance goal, namely the collection of bonus points for the other two players. That is, the social expectation that players will try to collect many bonus points for the other players will influence information driven decisions^23^. Importantly, when players decide against the majority, motives underlying such a choice differ between norms. Where a norm prompts a decision with the majority, then the decision against the majority is violating the current norm. By contrast, under a normative prompt to decide against the majority, decisions against the majority will be in line with the norm. To embed player’s decisions in a real social context, we openly declared how many bonus points each player actually collected for the others at the end of the experiment (which was known to players beforehand).

We investigated the interaction between normative and informational influences on a behavioural and at a neural level using functional magnetic resonance imaging (fMRI). We specifically asked, how are choices under simultaneous presentation of social and perceptual information influenced by normative influences. We expected that participants, as in previous experiments^6^, would increase their performance through integration of social and perceptual information^7^. We also expected that normative prompts to either follow, or go against, the majority decision would modulate this balance. Crucially, as individuals often follow social information to optimize their responses, particular decisions against the majority should provide mechanistic insights into norm compliance.

## Results

We independently manipulated information at two levels. Firstly, dot movement coherence determined the reliability of participants’ perceptual information. Secondly, social information (depicted as arrows on top of the moving dots) was either valid, invalid, or devoid of any informational value (arrows pointing in different directions). Additionally, participants were presented with three prompts on how they could obtain bonus points *for the other two participants* in a session. In a baseline condition (NONE), no bonus points were obtained. In the SAME condition, participants collected bonus points if they decided in the same direction as the other two players, conditional on all three participants being correct. In the ONLY condition, participants collected bonus points when deciding correctly against the congruent opinion of the other two players. Players received points for correct choices. Bonus points allocated to the other players had no impact on the remuneration of the index player.

### Effect of social information and norms on accuracy

To pursue an optimality goal, players should aspire to increase their overall correct choices to collect as many points as possible by using the available perceptual and social information. Under conditions without social information, players only marginally increased their accuracy when dot coherence increased (accuracy dot coherence 0.1: 65% ± 0.13%; dot coherence 0.15: 69% ± 0.15%; paired Wilcox rank sum test; N = 59; W = 1424, p = 0.08). As social information is often reliable, we manipulated social information such that it was more often valid (38.5%) than invalid (23%). Under conditions without normative influences, players increased their overall correct choices significantly through the use of social information compared to the no social information condition by 4.7% (±0.11%; Wilcox signed rank test; N = 59; V = 516; p = 0.005), thus using social information to their overall advantage. This difference was no longer present in the SAME condition (0.01% ± 0.09%; Wilcox signed rank test; N = 59; V = 819; p = 0.6) due to a large number of incorrect choices under invalid social information (see below). In the ONLY condition, there was a slight decrease in correct choices by 3.6% (±0.11%; Wilcox signed rank test; N = 59; V = 1148.5; p = 0.047).

If players used social information validity of this information should have a direct influence on players’ accuracy. We tested for this influence on accuracy via a linear mixed model that included social information validity, coherence of dots, social norm, and their interactions. We found an increase in the number of correct choices when social information was valid compared to a no social information condition. Likewise, a performance decrement was seen when social information was invalid (main effect validity of social information: *F*_1,638_ = 239.9, p < 0.001; Fig. 2A). This bidirectional effect was stronger in the SAME condition. In the ONLY condition, the effect of social information on performance was attenuated such that players performed at a level similar to a no social information condition (Interaction validity of social information and norm: F_2,638_ = 81.9, p < 0.001; Fig. 2A). We could not detect any influence of the coherence level on player’s accuracy (main effect and interactions all p > 0.25). We obtained a similar result, if analyses were restricted to players with high accuracy (>75% correct; Figure S4) and in a control experiment where information in phase two reflected actual decisions of the other two players from phase one and was thus not experimentally manipulated (Figure S2,; Table S2).

**Figure 1:**
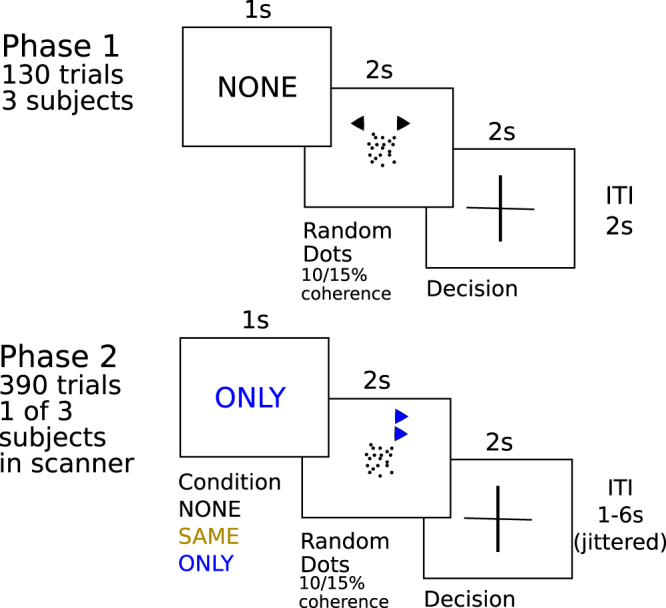
Experimental setup. Participants had to judge the direction of motion in a random dot kinematogram (*perceptual information, phase one*). In *phase two*, in addition to viewing the kinematogram they were also shown the decisions of two other players (*social information*), where they were told these responses were taken from these individuals *phase one* responses. In *phase two*, each correct response was rewarded by one point. Crucially, in this phase players could collect bonus points for the *other* two players, thereby adding to these players’ remuneration (but not their own). In brief, when prompted with the word SAME, they earned bonus points if deciding in the *same* direction as the other two players, conditional on all players being correct. When prompted with the word ONLY, they could earn bonus points for the other players by deciding *against* the concordant choice of these players, conditional on this decision being correct (*normative influence*). Players received no feedback on choice accuracy.

**Figure 2:**
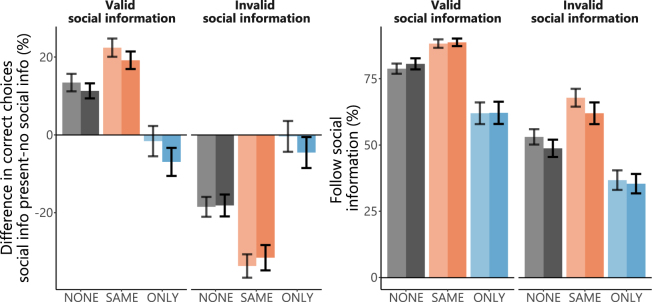
Behavioural Results. (**A)** Players (N = 59) used social information and consequently were more often correct when social information was valid than under a no social information condition (NONE, grey bars). When social information was invalid the proportion of correct choices made by the player decreased (NONE, grey bars). These effects were stronger for the SAME condition for both valid and invalid information (orange). In the ONLY condition (blue), correct choices were similar to that of the no information condition (players deciding in different directions). (**B)** Players followed social information more frequently in the SAME condition and less often in the ONLY condition compared to the NONE condition. In both panels: Shading indicates different coherence levels in dot motion with lighter shading indicating 10% coherence, darker shading 15% coherence. Error bars denote standard error of the mean. For statistics see main text.

We found significantly longer reaction times (calculated as the difference between trials with social information compared to no social information) when invalid social information was presented compared to valid social information (M_valid_ ± SD = 0.63 ± 0.14s; M_invalid_ ± SD = 0.68 ± 0.16s; Wilcox signed rank test (paired); V = 457, p = 0.001; Figure S1).

### Effect of norms on propensity to follow social information

In a next step, we assessed how norms and validity of social information influenced players’ choices. We expected players’ decisions in line with social information (i.e. conformity) to be influenced by our experimental manipulation of norm prompts. This was indeed the case, as a prompt to decide with the social information (SAME) increased choices in line with the social information under valid and invalid social information. A normative prompt to decide against the social information (ONLY) had the opposite effect and decreased choices in line with social information (main effect norm condition: χ^2^ = 459.0, df = 2, p < 0.001; main effect validity social information: χ^2^ = 480.8, df = 1, p < 0.001; Fig. 2B). In contrast to accuracy, coherence level influenced choices in line with social information when social information was valid (interaction validity of social information and coherence level: χ^2^ = 7.39, df = 1, p < 0.01). That is, players followed invalid social information less when perceptual information was more reliable, i.e. dot coherence was increased.

### Informational influences on decisions in functional magnetic resonance imaging (fMRI)

Our experimental design involved two main manipulations: 1. Adding social information to the perceptual information. 2. Adding norms as prompts to decide with or against the provided social information. We used fMRI data to investigate these effects during stimulus presentation. Behavioural data suggests that players used the social information and that the addition of social information influenced accuracy. To investigate this effect of added social information, we compared blood oxygen level dependent (BOLD) responses between conditions with coherent social information (social information present) contrasted with incoherent conditions (no social information). Occipital areas showed a relative decrease in activity when social information was present compared to no social information conditions (see Table S2).

### Normative influences on decisions in fMRI

**Figure 3:**
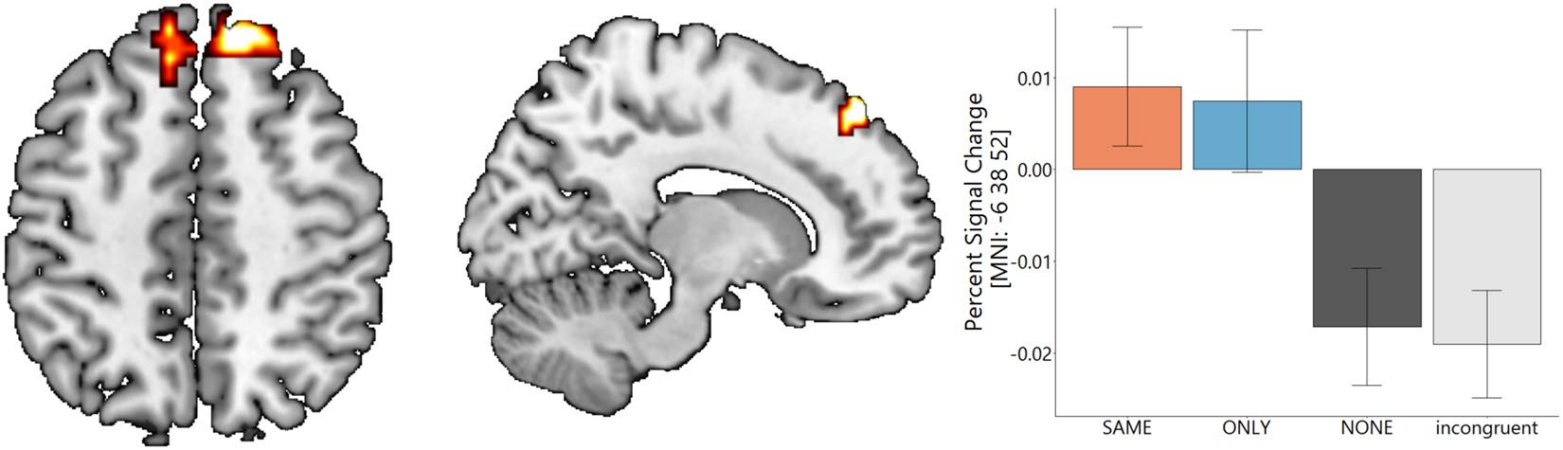
Dorsomedial prefrontal cortex was jointly activated in norm conditions (SAME and ONLY weighted equally) compared to a no norms condition under congruent social information during dot motion. Contrast thresholded at p < 0.05 FWE corrected on the cluster level and p < 0.001 on a voxel level. We compared percent signal change (extracted via a 6 mm sphere at MNI: x = −9 y = 23 z = 64) to the incongruent information condition, and this enabled us to show that the increase in activity in both norm conditions exceeds activity in the incongruent condition and the no norm condition (for detailed activations see Table S5).

**Figure 4:**
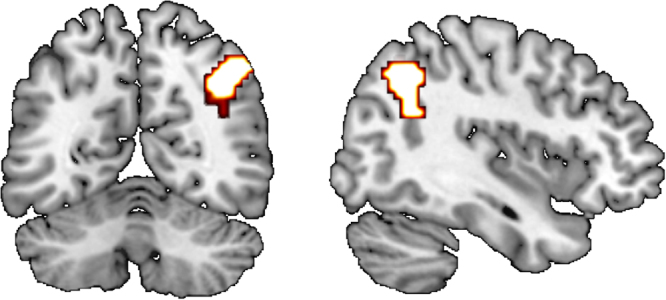
Right angular gyrus (MNI x = 39 y = −58 z = 50) showed increased functional connectivity with dmPFC under normative influences compared to conditions without norms (NONE) as indexed by a psychophysiological interaction (BOLD signal in seed region interaction with normative prompt present vs not present). Seed region at MNI x = −9 y = 23 z = 64 with 6 mm sphere. Contrast thresholded at p < 0.05 FWE corrected on the cluster level and p < 0.001 on a voxel level.

Behaviourally, different norms (SAME and ONLY) had distinctive modulatory effects on social information processing: SAME increasing the reliance on social information and ONLY decreasing it. Using fMRI data, we next compared conditions involving normative prescriptions to a no norm condition to obtain an estimate for the joint effect of norms. During stimulus presentation, dorsomedial prefrontal cortex was more engaged for norm conditions compared to no norm conditions (Fig. 3, Table S3). Activation of the thalamus was also seen at a more lenient voxel level threshold of p < 0.005 (Table S3). When testing for differences between the two norm prompts, we could detect no suprathreshold activation for a contrast between SAME and ONLY condition. To identify regions that were functionally connected under normative influences to this prefrontal region, we conducted a psychophysiological interaction. We calculated a separate GLM with the deconvolved time series from the medial prefrontal cortex, a psychological variable indicating whether norms (SAME, ONLY) were present in a given trial, and the respective interaction. Testing for the effect of this interaction, we found that a region in right angular gyrus, extending into supramarginal gyrus showed enhanced functional connectivity with dorsomedial prefrontal cortex under social norms compared to the condition without norms (NONE) (Fig. 4). Again we could not detect any suprathreshold effect that was specific to either the SAME or the ONLY condition.

### Deciding against social information under normative influences

Participants in our experiment exploited social information and increased their overall accuracy in the no norms condition indicating that following social information is the preferred decision. Deviating from this decision, constituted the crucial difference between the two norm prompts. In the SAME condition, deciding against the social information was associated with a norm violation. In the ONLY condition, the same decision against social information resulted in a norm compliance. These two conditions thus yield important insights how information and norms interact. To investigate this, we parametrically modulated each condition regressor with participants’ responses using a *with* vs *against* social information comparison.

**Figure 5:**
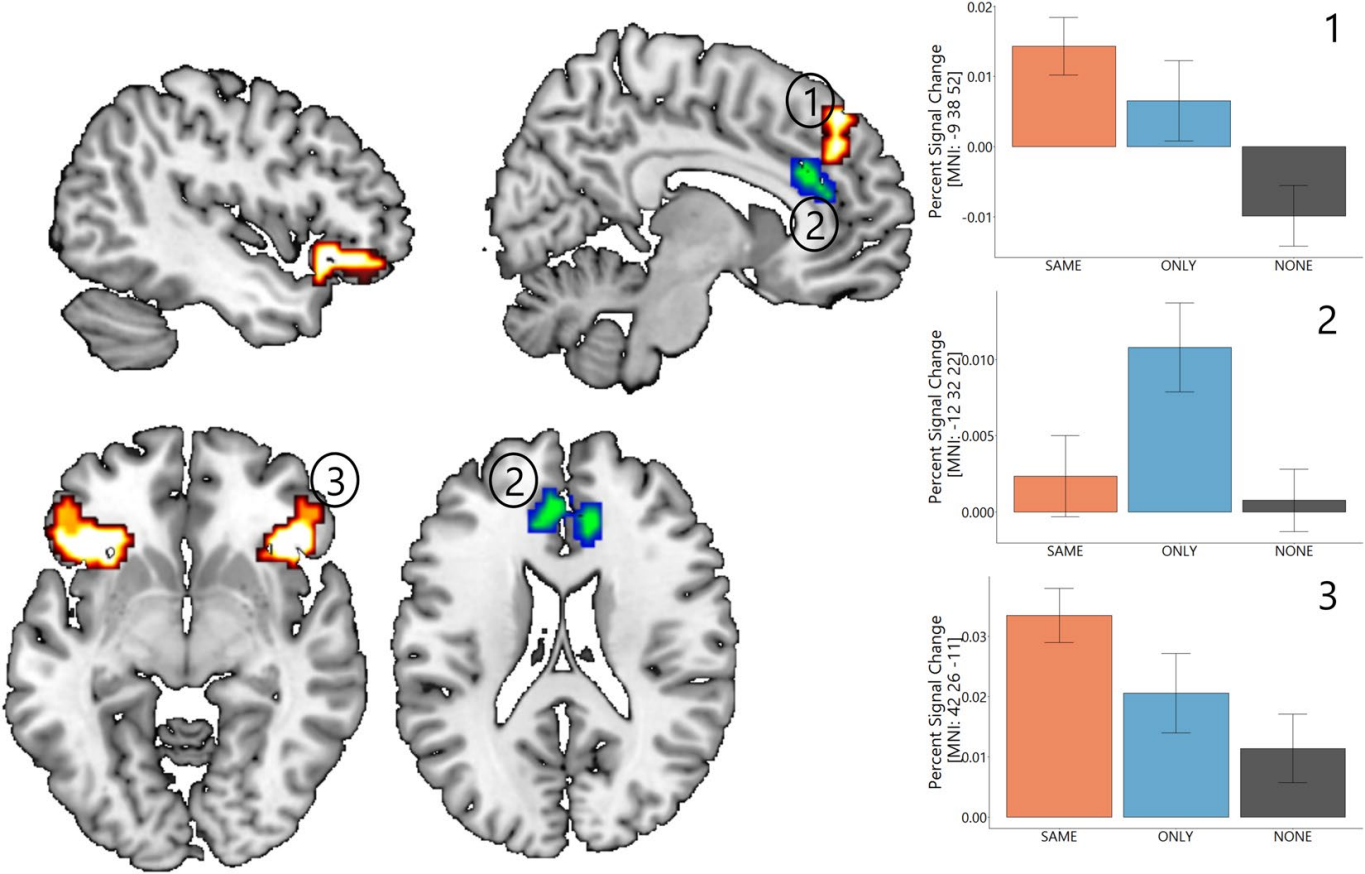
Parametric modulation when deciding *against* social information compared to deciding *with* social information during stimulus presentation in the two norm conditions. In the SAME condition (white/red) posterior medial frontal cortex (1) and bilateral inferior frontal gyrus (3) were activated. In the ONLY condition (green/blue) anterior cingulate cortex (2) was engaged. All contrasts significant at p < 0.05 FWE corrected on a cluster level with a voxel level threshold of p < 0.001. Percent signal change was extracted with a 6 mm sphere at the given MNI coordinates for the parametric modulator in each condition to allow for effect size comparisons across conditions.

In the SAME condition, players that decided against the social information also decided against a normative prescription to follow the others. During stimulus presentation, activations in posterior medial prefrontal cortex, bilateral vlPFC extending into anterior insula were increased (Fig. 5, Table S4, p < 0.05 FWE corrected on a cluster level with a voxel level threshold of p < 0.001).

In the ONLY condition, players that decided against the social information complied with a normative prescription to decide against the others. During stimulus presentation, anterior cingulate cortex activity increased when deciding against social information (Fig. 5, Table S5, p < 0.05 FWE corrected on a cluster level with a voxel level threshold of p < 0.001). For the no norm condition, we could not detect any activations above threshold when players decided against social information.

## Discussion

Our behavioural results show that experimentally applied prosocial norms that have consequences for others’ remuneration, but are independent of a player’s own rewards, influence choice behaviour even though informational components were equivalent across norm conditions. Players generally use social information to their benefit when no external rules apply, confirming findings from previous work^6,7,21,24^. Social information reliance increased even further if other players are potentially rewarded for a unanimous and correct decision. By contrast, players no longer bias their decisions towards social information if bonus points are available when deciding against the other two players. That is, both condition norms elicit behavioural change in line with a *compliance goal*. Whereas in the SAME condition players increased their reliance on social information, they disregarded social information in the ONLY condition. Notably, in both normative conditions the total number of correct choices was reduced compared to a no norms condition indicating that players deviated from following an *optimality goal*.

A bidirectional normative effect on choice behaviour is underpinned by differential brain activation in the two norm conditions compared to the no bonus point condition. To investigate these effects at a neural level, we first examined the effect of social information on decisions. Here, occipital regions showed a decreased activity when social information was present, possibly indicating less attention was allocated to the dot motion in these trials^25^. In the two norm conditions, players had to reconcile informational influences, dot motion and social information, using a rule that enabled them to obtain bonus points for the other players. In the norm conditions, this was associated with enhanced activity in the dorsomedial prefrontal cortex (dmPFC). Among the roles ascribed to dorsomedial prefrontal areas are a monitoring of ongoing behaviour including a flexible adaptation to current demands^26–28^. Dorsomedial prefrontal cortex in combination with thalamic activation (detected in our experiment only at a lenient threshold) also has a role in exerting cognitive control over action selection^29,30^, thought to be established via cortico-striatal loops that dynamically engage inhibitory control^30–33^. Interestingly, in the context of our findings, blockade of the dorsomedial prefrontal area via transcranial magnetic stimulation is reported to reduce conformity behaviour^34^. Our findings support a view that dorsomedial prefrontal cortex is important for modulating responses that are in line with higher order goals, in our case a compliance goal that considers other’s outcome^35^.

The area in dmPFC showed increased functional connectivity to the right angular gyrus, a region involved in multisensory integration^36^, when normative prompts were present compared to a condition without normative prompts. We could, however, not detect any increased activity in the right angular gyrus specific to the normative conditions (e.g. in the contrast norms - no norms). This could indicate that angular gyrus does not play a specific role in the integration of normative influences with informational influences, but instead serves as an integrative hub at the informational level. Under normative influences, angular gyrus might engage dmPFC to regulate a need for response adjustments due to conflict between informational and normative goals. An alternative explanation comes from the literature that assigns a role for the angular gyrus in mentalising about others^37,38^. Here dmPFC and angular gyrus/SMA comprise a mentalising network that is engaged when reasoning about mental states of others^39,40^. In our experiments, when social norms were present, this network was activated as dmPFC angular gyrus functional connectivity increased. Thinking about the consequences for the decision of others might have elicited such a neural response. It is, however, in our experimental paradigm difficult to disambiguate an information integration from a mentalising perspective as ventral parietal cortex is involved in multiple cognitive processes such as perceptual reorienting and theory of mind^38^.

A conflict between available information and norm prompt is resolved differently in the two norm conditions, which is reflected in observed choice differences. As participants followed social information on a majority of trials, a decision against the majority denotes an effortful response. In the SAME condition, where there is a strong informational and normative prior to decide with the social information, responses in the direction of social information were overruled only in a small number of trials, possibly when perceptual evidence was particularly high. In such a case, ventrolateral prefrontal cortex has been reported to be activated^41–43^. This stimulus driven inhibition of a dominant response tendency has similarities to that seen in stop signal tasks^44^. By contrast, dorsal anterior cingulate cortex (dACC) was engaged during dot motion when deciding against social information in the ONLY condition. Here, participants need to monitor conflicts between informational and social information and adjust their behaviour accordingly, a function putatively subserved by dACC^45–47^.

Perceptual decision-making in our experiment is thus influenced by norms such that congruent social information induces a response bias in line with the social information. We show that this response bias is strengthened when optimality and compliance goal mandate the same response. In this case, a response against the social information is putatively elicited through stimulus driven response inhibition. When the compliance goal and the optimality goal conflict, response biases are attenuated and participants flexibly adjust their behaviour under consideration of both goals. Importantly, we show that the same response, deciding against social information, is underpinned by different activation patterns that are a direct consequence of whether norms conflict or concord with decision relevant information.

There are a number of limitations that apply to our findings. Whereas the sample size for the behavioural study (N = 59) is within standard limits for these types of studies, the fMRI analysis is only conducted on a subset of N = 17 participants. As we report whole brain corrected analyses that are based on a p < 0.001 uncorrected voxel threshold it is possible that these results might not generalize to the population at large. In other words, the low sample size precludes estimating individual differences where social preferences vary between individuals or between men and women. Due to low power it is entirely possible that we have missed real differences, for example in the contrast between the two social norms where we did not detect suprathreshold differences.

Our findings go beyond recent accounts in which conformity behaviour depends on reinforcement learning mechanisms akin to those underlying asocial learning^34,48^. Note that we did not give feedback on the correctness of choices to participants. Under conditions where such feedback is available the reliability of social information could be learnt via reinforcement learning^8^. Previous experiments have investigated conformity (i.e. decisions in line with a majority) but here we investigated anti-conformity situations, including how these guide choices with or against reliable (social) information. A possibility suggested by our MRI data is that under conditions where normative influences conflict with available information close monitoring of a possible conflict during stimulus presentation can elicit a response against the social information. Under conditions where norms mandate a decision in line with social information it potentially becomes clear only at the end of the stimulus presentation that social information is invalid. Here, a fast response inhibition of customary choices is necessary.

Our findings inform interpretations of classical findings from social psychology. A decision against social information under social normative influences has echoes of the famous Asch line experiment where individuals conformed with an incorrect unanimous majority opinion on critical trials^49^. Contrary to critical trials, compliance and optimality goals coincided in many trials preceding critical choice trials. That is, both confederates and focal participant decided correctly and social information was highly reliable. The conformity responses observed in the Asch experiment are thus comparable to our experiment where participants were confronted on particular trials with invalid social information and a normative prompt to decide with the majority. In our account, conformity responses in such a case fail to elicit a reactive response inhibition. A direct comparison to a stop signal task could allow identification of whether the underlying neural mechanisms are similar and or whether unique circuits recruited under normative influences.

## Methods

Participants (N = 59; 33 female; mean age = 28) were recruited from the local participant pool and were paid on average 29 Euro, consisting of a basic amount, a bonus for correct answers, and bonus points collected by the other players (see below). All participants entering the scanner (n = 24; 12 female) were right-handed and had normal or corrected-to-normal vision. Participants reported no previous history of neurological or psychiatric illness. Before beginning, written instructions were given and written informed consent was obtained from all participants. All procedures were approved by the ethics committee of the psychology department at Humboldt Universität Berlin (proposal number: 2014_15). All methods were performed in accordance with the relevant guidelines and regulations.

### Experimental task

In the experimental task, three participants per session decided whether dots in a random dot kinematogram task (RDK) were moving to the left or right (Fig. 1). In case one participant did not show up (5 sessions), we had a student assistant on stand-by that acted as a stooge. The experiment was split into two phases with 130 trials in phase one and 390 trials in phase two. The first phase was to familiarise participants with the setup. In the second phase, we added social and normative information to this basic design. In phase two, one of the participants performed the task inside the MRI scanner.

In each trial during phase one, participants first saw the word ‘NONE’ in the centre of the screen for 1s (Fig. 1). The RDK was then displayed for 2s followed by a 2s interval where participants decided to which side dots were moving. Responses during stimulus presentation were not recorded. Difficulty of the task was manipulated by varying the percentage of the dots moving consistently in one direction (10% and 15%, presented equally often). During stimulus presentation, two arrows were displayed on top of the dots that were pointing either in the same or in opposite directions. Arrow directions in phase one were random and not connected to the direction of the dots as was explained to participants. A fixation cross in the middle of the screen appeared after the response. Participants received no feedback during the whole experiment whether their decision was correct. Before a new trial started, there was a jittered 2 to 6s inter-trial interval.

### Social information

For phase two, we informed participants that we had collected their decisions during phase one and that the decisions of the other two participants would be displayed as arrows on top of the RDK. Each correct decision regarding dot movement in phase two yielded one point for the player. Participants played a total 390 trials, from which 38.5% were valid or no-information (arrows in different directions) each and 23% were invalid. We reduced the number of invalid social information trials as it is less likely in a 2AFC task that two players will jointly decide in the wrong direction than the correct direction given that single player accuracies exceeded 50% (i.e. random choice). No player actively raised suspicions during and after the experiment about this manipulation. We did, however, not ask participants whether they assumed social information to be rigged. Moreover, we conducted a behavioural control experiment (N = 59) where we presented players with the actual information of the other players from phase one with results largely in line with the experimental findings of our main experiment (see Figure S4).

### Normative information

Normative information was operationalised as bonus points (worth 1 Cent) that players could collect for the other players (but not for themselves). In the SAME condition, participants were presented with the word ‘SAME’ (yellow colour) and in the ONLY condition with the word ‘ONLY’ (blue colour) at the outset of the trial. In the SAME condition, participants collected bonus points if they decided in the same direction as the social information, given that both arrows point in the same direction, and all three players were correct in their estimate. In the ONLY condition, participants could collect bonus points if they decided against the social information, again both arrows in the same direction, and their estimate was correct. In trials that were indicated by the word ‘NONE’, participants could not collect bonus points (NONE condition). Beyond the bonus points, players could receive points for themselves when deciding in the correct direction. We ensured that participants understood the rules properly by asking the group after phase one several questions regarding their correct understanding of the bonus points. Importantly, we told participants that the players would come together again after phase two and we would openly announce the bonus points collected by each player to all players to make their decisions salient in a social context. Events were randomised with a restriction to a maximum of three identical conditions in succession.

### Analysis of the Behavioural Data

We analysed the difference in proportion correct between the NONE, SAME, ONLY and the condition without social information depending on condition, coherence level, and validity of social information via linear mixed models using the lme4 package^50^ in R^51^. We assumed a random effect structure where player identity had an influence on the intercept. Significance tests were conducted using the Kenward-Rogers correction^52^ for degrees of freedom in the afex package. We modelled choices in line with social information (coded as 0/1) with a generalised linear mixed model assuming a binomial error structure. We used condition, validity of social information, and coherence as dependent variables with a random effect structure that modelled player identity as an influence on the intercept, and assessed significance via likelihood ratio tests. This was conducted as an F-test in the case of a linear mixed model (difference in accuracy to baseline with normally distributed errors) and a Chi-square test in case of a binomially distributed variable (choosing in line with social information). Throughout the manuscript, we give mean values and report standard errors of the mean. For simple comparison of accuracy values (proportions) and reaction times, we used non parametric tests as both variables are not normally distributed (reaction times are assumed to be gamma distributed and proportions are binomially distributed).

### MRI data

#### Image acquisition and analysis

Whole brain T2*-weighted echo-planar imaging BOLD fMRI data were acquired from 24 participants with a Siemens Trio 3 T (Siemens Medical Solutions, Erlangen, Germany) magnetic resonance scanner using a 12-channel head matrix coil, with 33 slices recorded in descending order (64 × 64 voxels; resolution 3 × 3 × 3.5 mm slices), a volume repetition time (TR) of 2000 ms, an echo time of 30 ms. The fMRI data were preprocessed and analysed in an event-related manner with SPM12 (v7219) software (Wellcome Trust Centre for Neuroimaging, London, UK).

Preprocessing consisted of unwarping through a field map, spatial realignment, co-registration to the participants’ T1 image, normalization to Montreal Neurological Institute coordinates via the new segment procedure in SPM12, and spatial smoothing with a Gaussian kernel with a full width at half-maximum of 8 mm. We estimated a GLM on the first level with each condition with coherent social information (NONE, SAME, ONLY) as a separate regressor and one regressor for all trials with incoherent social information. We added five parametric modulators to each regressor that coded for (i) whether players decided with or against social information, (ii) whether the congruent social information was true or false, (iii whether a decision was correct or incorrect, (iv) coherence level, and (v) whether players pressed left or right. For the incongruent condition, we omitted the first two regressors as they were not applicable. Parametric modulators were not orthogonalised. We estimated a GLM that investigated BOLD signal during evidence accumulation process with the onset at stimulus onset and a boxcar function of length 2s (stimulus length). Additionally, we added six head motion regressors to account for systematic variance caused by participants’ head motion. Trials were split evenly into two sessions. In between sessions, we recorded a T1 structural image used for normalisation. We had to exclude one participant from the MRI analysis since accuracy in the random dot task dropped significantly below random choice in the no social information condition in phase 2. It was thus unclear whether this player understood the experiment and particularly the buttons on the response box correctly. A further five participants were excluded from the MRI analysis because they did not, or only very infrequently (<5 times in 80 trials), decided against the SAME norm. Without decisions against the norm, it is not possible to investigate the interaction between informational and normative influences. In particular, the parametric modulator of interest that models changes in BOLD amplitude, based on whether players decided against or with social information, could not be estimated in these instances. Seventeen participants (9 female, mean age = 29 ± 4.6) thus entered the final fMRI analysis.

### PPI Analysis

We performed a functional connectivity analysis by analysing the psychophysiological interaction between activity in the dmPFC and a psychological factor, normative prompt present/not present. One regressor in our PPI analysis represented the deconvolved activity from a VOI at MNI coordinates x = −9 y = 23 z = 64 with a 6 mm sphere. We also entered a regressor where we coded whether normative prompts were present or absent. Our final regressor of interest was the interaction between the previous two regressors.

### Data availability

Behavioural data, analysis script for the behavioural data, and unthresholded t-maps for fMRI contrasts can be found under osf.io/7zn62.

## Electronic supplementary material

Supplementary Material
