# Supplementary material for: Neural substrates of norm compliance in perceptual decisions
Source: Sci Rep. 2018 Feb 20;8:3315. doi: 10.1038/s41598-018-21583-8 (PMC5820261; doi:10.1038/s41598-018-21583-8)
Supplement: Supplementary file 1 — Supplementary Material [file 41598_2018_21583_MOESM1_ESM.doc]

**Supplementary Material accompanying the paper**

*Neural substrates of norm compliance in perceptual decisions*

Toelch, U., Pooresmaeili, A., and Dolan, R.J.

**Additional results**

**Analysis Reaction Times main experiment**


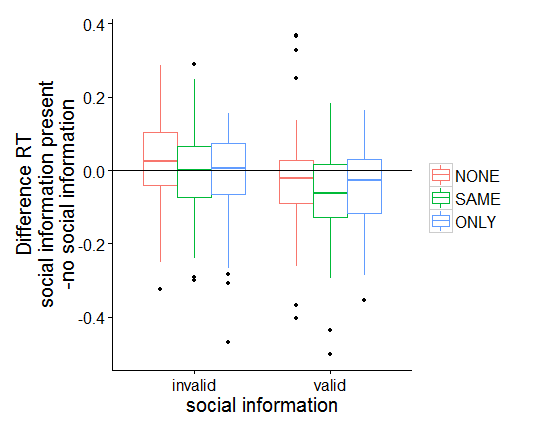


Figure S1: Difference in reaction time with social information present minus no social information (y axis in s) depends on whether social information was valid or invalid (x-axis). A model that accounts for repeated measurements and corrects for individual accuracy is presented in Table S1.

**Choice Behaviour in Pilot Study**

Prior to the main experiment, we conducted a pilot study to ascertain that behavioural effects of the norm conditions were present when displaying actual player decisions and not information optimised for the MRI experiment. In the pilot study, we did not include a condition without norms (NONE). 59 participants took part in this study and results are similar to the main study. In the SAME condition, participants clearly decide in the direction of social information (Figure S2). That is, players followed social information and this resulted in an increase in correct decisions when social information was valid and a decrease in correct decisions when social information was invalid. In the ONLY condition, this effect was attenuated and players had similar performance levels when compared to the no information condition.


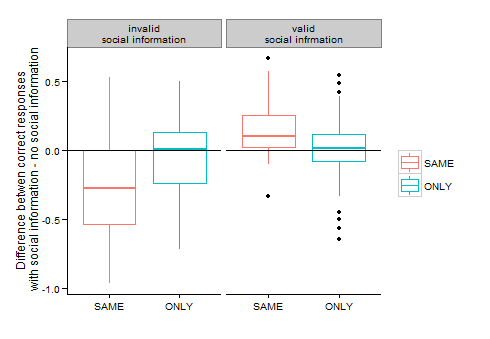


Figure S2: Experimental control with social information actually generated by the other players. In the SAME condition players reduce their correct choices under invalid information and increase their performance under valid social information. In the ONLY condition this differential change in performance is absent (for a statistical model see S2).

Table S2: Control experiment with actual social information by other players. Linear mixed model with correct choices as dependent variable. Independent variable are coherence, displayed norm, and validity of social information.

| **Coefficient** | **Estimate** | **Standard error** |
| --- | --- | --- |
| Intercept | 0.72 | 0.18 |
| Coherence | 2.66 | 0.58 |
| Trial Type: ONLY | 0.01 | 0.07 |
| Invalid Social Info | -1.4 | 0.12 |
| Valid Social Info | 0.95 | 0.08 |
| Interaction ONLY: Invalid Social information | 1.2 | 0.17 |
| Interaction ONLY: Valid Social information | -0.94 | 0.1 |

**Analysis restricted to high accuracy players**


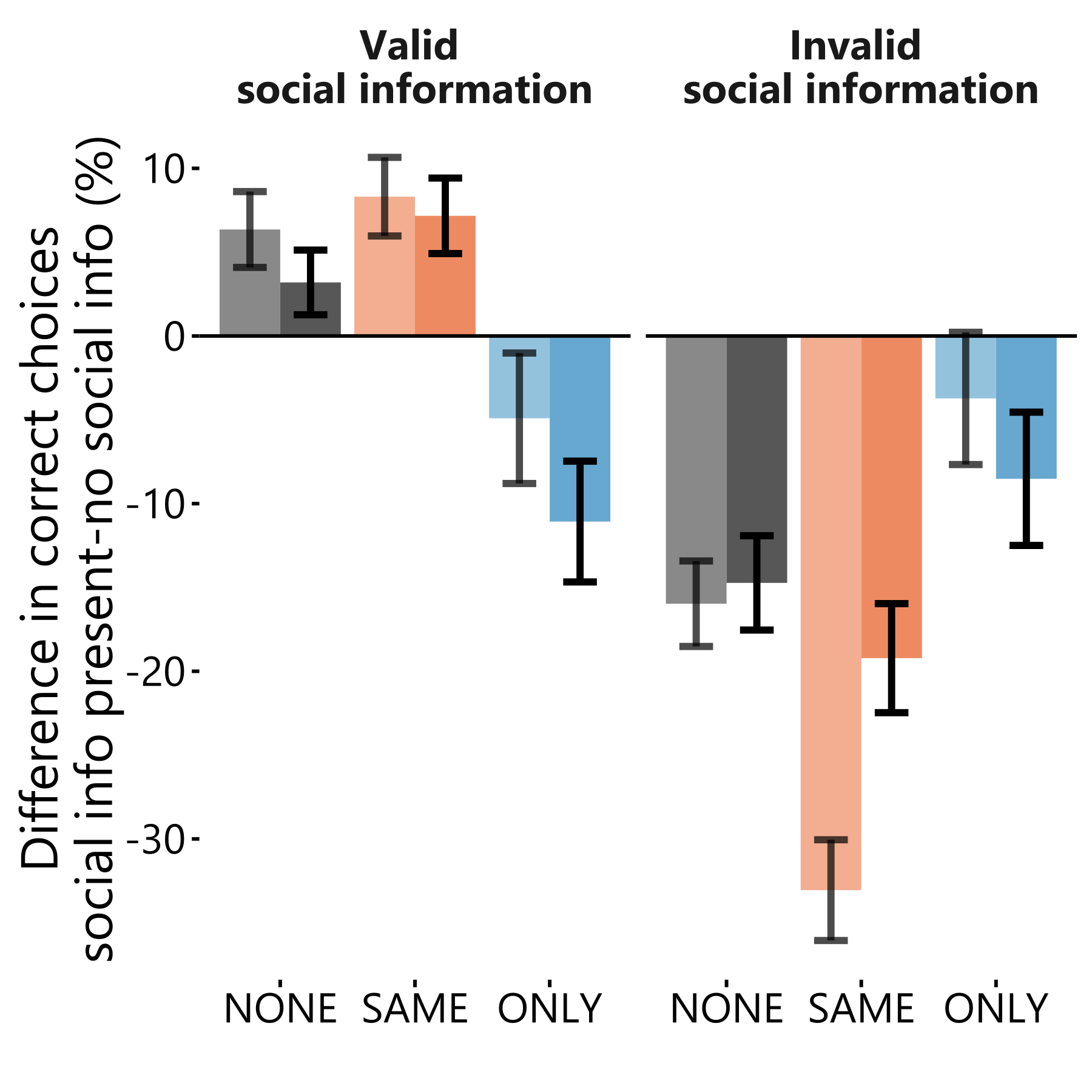


Figure S3 Restricting analyses to players that had high accuracies (>75% correct choices without social information) resulted in a similar behavioural pattern as in the whole data set (compare to Figure 2A in main manuscript).

**Additional MRI analyses and activation tables**

All anatomical labels have been retrieved using the anatomy toolbox in SPM.

Table S2 Coherent<Incoherent *during stimulus presentation* with coordinates in MNI space. Activations are whole brain family wise error corrected with a significance threshold of p<0.05 on the cluster level and a threshold of p<0.001 uncorrected on the voxel level.

| **Cluster**  **p (FWE-corrected)** | **Cluster Size** | **cluster p**  **(uncorrected)** | **x** | **y** | **z** | **Region** | **Side** |
| --- | --- | --- | --- | --- | --- | --- | --- |
| 0.005 | 170 | 0.001 | 24 | -88 | 4 | Middle Occipital Gyrus | R |
|  |  |  | 33 | -73 | 13 |  |  |
| 0.17 | 55 | 0.04 | -30 | -70 | 13 |  |  |
|  |  |  | -24 | -82 | 16 | Middle Occipital Gyrus | L |
|  |  |  |  |  |  |  |  |
|  |  |  |  |  |  |  |  |
|  |  |  |  |  |  |  |  |
|  |  |  |  |  |  |  |  |

Table S3 NORMS>NO NORM *during stimulus presentation* with coordinates in MNI space. Activations are whole brain family wise error corrected with a significance threshold of p<0.05 on the cluster level and a threshold of p<0.005 uncorrected on the voxel level. Bold faced activations are also significant at a more conservative threshold of p<0.001 uncorrected on the voxel level.

| **Cluster**  **p (FWE-corrected)** | **Cluster Size** | **cluster p**  **(uncorrected)** | **x** | **y** | **z** | **Side** | **Region** |
| --- | --- | --- | --- | --- | --- | --- | --- |
| 0.053026 | 234 | 0.004026 | 6 | -22 | 4 | R | Thalamus |
|  |  |  | -3 | -25 | 4 | L | Thalamus |
|  |  |  | 18 | -13 | 10 | R | Thalamus |
| **0.000524** | **585** | **3.87E-05** | 12 | 44 | 52 | R | Superior Medial Gyrus |
|  |  |  | -6 | 38 | 52 | L | Superior Medial Gyrus |

Table S4 Parametric Modulation Deciding against social information in the SAME condition *during stimulus presentation* with coordinates in MNI space. Activations are whole brain family wise error corrected with a significance threshold of p<0.05 on the cluster level and a threshold of p<0.001 uncorrected on the voxel level.

| **Cluster**  **p (FWE-corrected)** | **Cluster Size** | **cluster p**  **(uncorrected)** | **x** | **y** | **z** | **Side** | **Region** |
| --- | --- | --- | --- | --- | --- | --- | --- |
| 0.01 | 110 | 0.002 | 42 | 26 | -11 | R | Inferior frontal gyrus |
|  |  |  | 48 | 38 | -11 | R | Inferior frontal gyrus |
|  |  |  | 27 | 20 | -14 | R | Insula Lobe |
| 0.002 | 167 | <0.001 | -39 | 23 | -11 | L | Inferior frontal gyrus |
|  |  |  | -51 | 35 | -11 | L | Inferior frontal gyrus |
|  |  |  | -51 | 26 | -8 | L | Inferior frontal gyrus |
| 0.02 | 99 | 0.003 | -9 | 38 | 52 | L | Superior Medial Gyrus |
|  |  |  | 6 | 41 | 34 | R | Superior Medial Gyrus |
|  |  |  | -6 | 29 | 55 | L | Superior Medial Gyrus |

Table S5 Parametric Modulation Deciding against social information in the ONLY condition *during stimulus presentation* with coordinates in MNI space. Activations are whole brain family wise error corrected with a significance threshold of p<0.05 on the cluster level and a threshold of p<0.001 uncorrected on the voxel level.

| **Cluster**  **p (FWE-corrected)** | **Cluster Size** | **cluster p**  **(uncorrected)** | **x** | **y** | **z** | **Side** | **Region** |
| --- | --- | --- | --- | --- | --- | --- | --- |
| 0.05 | 76 | 0.009 | 9 | 32 | 22 | R | ACC |
|  |  |  | -6 | 38 | 19 | L | ACC |
|  |  |  | -12 | 32 | 22 |  |  |
